# APOE genotype, polygenic risk for Alzheimer’s disease, and plasma cytokines in participants of the multisite SuperAging Research Initiative

**DOI:** 10.1002/alz.095500

**Published:** 2025-01-09

**Authors:** Matt Huentelman, Ignazio Piras, Marcus Naymik, Bobbi Stark, Anna Bonfitto, Francis Taguinod, Rhiana Schafer, Adam Martersteck, Amanda Cook Maher, Changiz Geula, Emily J Rogalski, The SuperAging Research Initiative

**Affiliations:** ^1^ The Translational Genomics Research Institute (TGen‐ an Affiliate of City of Hope), Phoenix, AZ USA; ^2^ University of Chicago, Chicago, IL USA; ^3^ University of Michigan, Ann Arbor, MI USA; ^4^ Northwestern University Feinberg School of Medicine, Chicago, IL USA

## Abstract

**Background:**

The SuperAging Research Initiative (SRI) seeks to identify factors that promote cognitive healthspan. SuperAgers are defined as individuals aged 80 and older with episodic memory performance that is at least average for people who are two to three decades younger. A goal of the SRI is to identify the genomic contributors to SuperAging and herein we describe the interim results of DNA‐ and plasma protein‐based analyses.

**Method:**

Biospecimens were collected in concert with in‐person cognitive assessments. Genomic DNA (n = 125 SuperAgers) was isolated from PAXGene DNA tubes using a Promega Maxwell instrument. Genome‐wide SNP genotypes were generated using the GSA BeadChip (Illumina) and imputation was performed via TOPMed. Polygenic risk scores (PRS) for Alzheimer’s disease (AD) were generated using the summary statistics from the AD GWAS reported by Lambert *et al*. Plasma samples (n = 59 SuperAgers and 52 Controls) were analyzed using a Luminex panel that included 24 cytokine and inflammatory markers.

**Result:**

APOE allele and genotype frequencies were found to be similar to neuropathologically‐assessed control samples from the “TGen II” AD GWAS cohort (e4 allele frequency; SuperAgers = 9.8%. Controls = 10.8%). AD PRS distributions in the SuperAger samples were also similar to the TGen II Controls; however, three SuperAgers were noted to have AD PRS values that were significantly high risk. Two cytokines demonstrated association with SuperAging, IL‐7 (increased in SuperAgers; p = 0.003) and Interferon‐gamma (increased in SuperAgers; p = 0.04). However, after multiple hypothesis correction, neither remained significant.

**Conclusion:**

These results suggest that SuperAgers are not at lower risk for AD based on their APOE genotypes and AD polygenic risk scores. However, this does not account for all SuperAger participants as 15% are e4 allele carriers and 3% demonstrate AD PRS scores that are significantly elevated. Lastly, immune system profiling suggests that IL‐7 and Interferon‐gamma may be elevated in SuperAger plasma, suggesting that immune system function may be associated with SuperAging. This interim analysis of the SRI cohort supports the concept that the propensity to be a SuperAger is likely heterogenic. SuperAgers with high genetic risk for dementia may represent unique opportunities to search for resilience factors.

